# Detection of GM residues in foods derived from soy in Uberlândia - MG

**DOI:** 10.1186/1753-6561-8-S4-P127

**Published:** 2014-10-01

**Authors:** Marco Aurélio Bernardes Vieira, Mariana Menezes Nascimento Feldner, Aline Gomes Souza, Vivian Goulart-Alonso

**Affiliations:** 1 Universidade Federal de Uberlândia, Uberlândia, Brazil

## 

Soy is a product of great economic importance, since it covers a variety of marketing fields that permeate the human and animal consumption for the processed food industry. Thus, to maximize production and improve product quality, the legume is a frequent target for genetic modification. The concern with the long-term effect of the transgenic product, beyond the consumer's right to know about the product you eat demanded a law regulating the presence of these products in the market. Existing legislation in Brazil indicates that rates of GM products, with more than 1% should be informed on food labels. To ensure compliance with the law are necessary protocols able to identify and quantify the waste. Therefore, this project aims to evaluate the protocols of DNA extraction by CTAB method proposed by Ferreira and Grattapaglia [1] and the method proposed by FERRARI et al [2], as well as detect PCR transgenic residues in processed foods derived from soybeans. After extraction, DNA was quantified by spectrophotometry and absorbance ratio 260/280 nm was analyzed to determine the quality of the samples. Furthermore, the technique was used to eletrofrese agarose gel to compare the quality of DNA samples of soybeans with fresh samples processed soybeans. The protocol for DNA extraction from FERREIRA & GRATTAPLAGLIA was more efficient compared to the yield of DNA extracted from different samples, but in relation to quality of DNA both protocols showed similar results. The detection of residues in transgenic samples showed that the PCR amplified only the lectin gene (endogenous), confirming that the samples contained soy, but did not amplify the exogenous gene, suggesting that the samples did not contain residues of transgenic.
